# Application of VirCapSeq-VERT and BacCapSeq In the Diagnosis of Presumed and Definitive Neuroinfectious Diseases

**DOI:** 10.21203/rs.3.rs-2675665/v1

**Published:** 2023-07-11

**Authors:** Abhilasha P Boruah, Adam Kroopnick, Riddhi Thakkar, Annie E Wapniarski, Carla Kim, Rachelle Dugue, Eileen Harrigan, W. Ian Lipkin, Nischay Mishra, Kiran T Thakur

**Affiliations:** Columbia University Department of Neurology; Columbia University Department of Neurology; Columbia University Mailman School of Public Health; Columbia University Department of Neurology; Columbia University Medical Center; Columbia University Department of Neurology; Columbia University Department of Neurology; Columbia University Mailman School of Public Health; Columbia University Mailman School of Public Health; Columbia University Department of Neurology

**Keywords:** Neuroinfectious Disease, VirCapSeq-VERT, BacCapSeq, High-throughput sequencing (HTS)

## Abstract

**Background:**

Unbiased high-throughput sequencing (HTS) has enabled new insights into the diversity of agents implicated in central nervous system (CNS) infections. The addition of positive selection capture methods to HTS has enhanced the sensitivity while reducing sequencing costs and complexity of bioinformatic analysis. Here we report the use of virus capture based sequencing for vertebrate viruses (VirCapSeq-VERT) and bacterial capture sequencing (BacCapSeq) in investigating CNS infections.

**Design/Methods:**

Thirty-four samples were categorized: (1) Patients with definitive CNS infection by routine testing; (2) Patients meeting clinically Brighton Criteria (BC) for meningoencephalitis (3) Patients with presumptive infectious etiology highest on the differential. RNA extracts from cerebrospinal fluid (CSF) were used for VirCapSeq-VERT and DNA extracts were used for BacCapSeq analysis.

**Results:**

Among 8 samples from known CNS infections in group 1, VirCapSeq and BacCapSeq confirmed 3 expected diagnoses (42.8%), were negative in 2 (25%), yielded an alternative result in 1 (11.1%), and did not detect 2 expected negative pathogens. The confirmed cases identified HHV-6, HSV-2, and VZV while the negative samples included JCV and HSV-2. In groups 2 and 3,11/26 samples (42%) were positive for at least one pathogen, however 27% of the total samples (7/26) were positive for commensal organisms. No microbial nucleic acids were detected in negative control samples.

**Conclusions:**

HTS showed limited promise for pathogen identification in presumed CNS infectious diseases in our small sample. Before conducting larger-scale prospective studies to assess clinical value of this novel technique, clinicians should understand benefits and limitations of using this modality.

## Introduction

High-Throughput Sequencing (HTS) is a promising approach in diagnosis of neurological infections, as up to 60% of meningoencephalitis cases remain undiagnosed after routine clinical testing.^1,2,3^ With the ability to test for a multitude of pathogens in a single assay without the need for specific targeted primers as used in consensus polymerase chain reaction (PCR) tests, HTS focuses on a broad-spectrum and multi-target diagnostic method.^4^ HTS has the potential to identify infectious agents that are beyond a clinician’s initial presumptive differential diagnosis, particularly if the organism is novel, or a re-emerging zoonotic pathogen or rarely attributed to CNS disease. This can be particularly useful in diagnosis in immunocompromised patients who are prone to CNS infections. However, HTS also has several limitations in clinical practice, including expensive costs, risk of environmental contamination, data from host genomic sequences, and limited availability across varied clinical-resource settings.^5^

In this study, we used HTS testing on cerebrospinal fluid (CSF) samples from patients across diagnostic categories, including those with unidentified pathogens thought to have neurological infections and those with definitive CNS infections diagnosed by routine hospital-based diagnostic tests. Specifically, we utilized two capture-based enrichment techniques for HTS; 1] for virus pathogen identification-VirCapSeq-VERT, a technology with approximately 2 million biotinylated probes covering 207 viral taxa known to infect vertebrates^6^, and 2] for bacterial pathogen identification - BacCapSeq, which contains 4.2 million biotinylated probes covering 307 bacterial species including all known pathogens infecting humans.^7^

## Methods

A retrospective chart review was conducted on a cohort of patients admitted with presumed or definitive neuroinfectious or neuroinflammatory conditions who underwent lumbar puncture and had their CSF samples bio-banked at New York Presbyterian-Columbia University Medical Center/Children’s Hospital of New York (NYP-CUMC/CHONY) between 2017–2019. Bio-banked CSF specimens which were stored at −80 degrees Celsius soon after CSF collection were then analyzed.

Patients were selected based on chart review from the electronic medical record (EMR). The protocol was approved by the CUIMC institutional review board and all appropriate consent was received from all included patients. Thirty-four samples were selected in three categories: (1) Patients with definitive CNS infection by standard hospital-based clinical testing, (2) Patients without definitive microbiological diagnosis via standard testing who met clinical criteria by Brighton Criteria (BC) for meningitis or encephalitis^8^, (3) Patients who neither received a definitive microbiological diagnosis nor met BC but had infection highest on the differential by primary clinical team. CSF samples were de-identified and transferred to the laboratory maintaining cold-chain for VirCapSeq-VERT and BacCapSeq analyses. RNA extracts from CSF were used for VirCapSeq-VERT and DNA extracts were used for BacCapSeq analysis. HTS analysis was completed as part of usual clinical care in the time period of admission or shortly after discharge. Laboratory teams running HTS were blinded to all clinical data.

## Nucleic acid extraction and VirCapSeq-VERT library preparation

AllPrep DNA/RNA Mini Kit (Qiagen, Hilden Germany) was used to extract 300ul of CSF collected from 34 patients, with 50ul eluted to isolate DNA and RNA, respectively. Negative controls included 2 CSF samples from healthy controls without any known infectious disease, and 3 buffer extraction controls (no template controls) for background reads filtration in further analyses. Individual VirCapSeq-VERT libraries were prepared using the Hyper Prep kit (KAPA Biosystems, Boston, MA, USA) and unique barcodes. RNA extract was DNAse treated (DNAse I, Ambion, Life Technologies) and first strand cDNA was generated using Superscript III and random hexamer primers for reverse transcription (Invitrogen, Life Technologies). Prior to the second stranded cDNA synthesis, the cDNA was treated with RNAse-H using random primer extension and Klenow enzyme (New England Biolabs, Ipswich, MA, USA). Fragmented segments of an average size of 200bp were generated using enzymatic shearing from the resulting double stranded cDNA preparations for HTS. (Kapa biosystems, Roche). These fragmented segments were purified using Agencourt Axyprep AMPure Beads^9^ and libraries were prepared using the HyperPlus Library Prep Kit following the manufacturer’s protocol. The final libraries were quantified using Agilent TapeStation System. The libraries were then pooled and hybridized with the VirCapSeq-VERT probe set prior to a final PCR and sequencing on Illumina HiSeq 4000 system.

## Library preparation and capture sequencing with BacCapSeq

Individual BacCapSeq libraries were prepared from the extracted DNA also using the Hyper Prep Kit (KAPA Biosystems, Boston, MA, USA). Extracted DNA was fragmented using enzymatic shearing to generate segments of an average size of 200bp followed by the same steps as VirCapSeq-VERT sequencing. The libraries were pooled and hybridized with the BacCapSeq probe sets followed by a final PCR and sequencing on Illumina HiSeq 4000 system.

## Data Analytics and Bioinformatics Pipeline

After VirCapSq-VERT and BacCapSeq sequencing on the Illumina platform, 100bp single end reads were generated, which were de-multiplexed using bcl2fastq software v 1.8.4 into individual samples for further analysis, Illumina adapter sequences were trimmed from the demultiplexed FastQ files using cutadapt (v1.8.3)^10^, quality filtered and end trimmed with PRINSEQ software (v0.20.3)^11^ and cleaned of human host sequences using Bowtie 2 mapper (v2.2.9)^12^. Assembly of quality filtered and host subtracted reads was performed using MIRA Assembler (v4.0). Assembled contiguous sequences and unique singletons were subjected to homology searches against the entire GenBank nucleotide database using Megablast.

All the contiguous sequences and unique singletons from assembly, which did not assign any hit with MegaBLAST, were subjected to NCBI blastx. Based on Megablast and blastx analysis viral and bacterial sequences matching with Illumina reads and contigs, were downloaded from NCBI and used for mapping to recover partial or complete genomes and determine genome consensus sequence and breadth of coverage. The Fastq reads were then imported into Geneious 10.0.9 (https://www.geneious.com) and the reads were trimmed to remove low quality sequences. The map-to-reference tool was used to assemble the reads using the reference genomes from GenBank.

## Results

Between November 2017 and October 2019, CSF samples from 34 patients admitted to the hospital between November 2017 and October 2019 were analyzed with VirCapSeq and BacCapSeq. Of these 34 patients, 58% (21/34) were female and 94.1 % were adults over 18 years old (32/34). Upon characterization of immune status, 35.3% (12/34) were immunocompromised, as defined by evidence of active cancer, active HIV/AIDS infection, organ or stem-cell transplant recipient, active use of immunosuppressant therapy or other primary immunodeficiency (CDC 2022). A majority of the patients had additional medical comorbidities upon presentation (31/34; 91.1%), and 38.2% (13/34) had previous neurological history. The most common presenting symptoms across all groups included altered mental status (19/34, 55.8%), followed by focal weakness (13/34, 38.2%), headache (10/34, 29.4%), and seizure (8/34, 23.5%).

## Group 1: Proven CNS infection by gold standard clinical testing

Within the definitive CNS infection group (n = 8), VirCapSeq-VERT and BacCapSeq confirmed presence of genetic material from three of the original results (Table 1) In one of these cases, Pseudomonas was identified in addition to the originally diagnosed Human betaherpesvirus 6B (HHV6B). All of these cases involved immunocompromised participants with active use of immunosuppressive therapy (steroids, CAR-T therapy). One of the definitive cases of Varicella Zoster Virus (VZV) identified alternative pathogens (GBV-C/ Human Pegivirus) and failed to align sequences to VZV. In the other definitive case of VZV antibodies positive, HTS accurately identified sequences of VZV in CSF and additionally found Torque Teno Virus. Both cases occurred in immunocompromised participants with histories of bone marrow transplantation and HIV, respectively. The remaining four definitively diagnosed samples (4/8; 50%) were negative. One of these participants had a definitive diagnosis of Cryptococcus neoformans by antigen test originally, therefore the negative reading by VirCapSeq-VERT and BacCapSeq was as expected. Additionally, another sample had an initial positive read for Borrelia burgdorferi on serological testing, thus the negative read on sequencing was also expected given this result was likely captured later in infection when the nucleic acid has been degraded. The additional two samples had original diagnoses of JC Virus and HSV-2 per standard clinical testing, however VirCapSeq-VERT/BacCapSeq yielded no sequences for these agents. A majority of the patients in this cohort were immunocompromised (6/8, 75%) and 4/6 (67%) of them had a pathogen detected on VirCapSeq-VERT/BacCapSeq. 50% (3/6) of these samples analyzed confirmed the original diagnosis based on gold standard testing.

## Groups 2 and 3: Suspected CNS Infections

Of the suspected CNS infection patient group, 6 were designated as group 2 according to Brighton criteria classification for meningitis/encephalitis, and 20 samples that did not meet Brighton criteria were designated as group 3. Four of six (67%) of the group 2 cases were positive for a pathogen, while 7/20 (33.3%) of samples in group 3 were positive. Overall, 11/26 (42%) of the samples were positive for at least one pathogen by VirCapSeq-VERT/BacCapSeq analysis. Of the positive illumina reads, 7/11 (64%) had commensal organisms (Bacteroides, Herbaspirillum, Faecalibacterium, etc) as at least one of the detected pathogens on HTS.

In group 2 all patients were immunocompetent. Of the 4/6 samples with positive reads on VirCapSeq-VERT/BacCapSeq, identified pathogens included Herbaspirillum, Bacteroides, Faecalibacterium, Bifidobacterium, Staphylococcus, Streptococcus, Neisseria, Leclercia, Burkholderia, and Pseudomonas. None of the positive pathogens significantly correlated with clinical suspicion diagnoses formulated by the care team (Table 2).

Within group 3, 6 samples (30%) returned one bacterial species, while 1 returned multiple (>2) bacterial species. Two (10%) samples were positive for viral pathogens. In the case of the viruses, one sample originated from a HIV + patient which returned positive for HIV on HTS testing. The second viral case returned hepatitis E. 15 samples (57.7%) were negative for any pathogen on HTS testing. The most commonly identified pathogens in these cohorts were Bacteroides (3/11), Herbaspirillum (2/11, and Faecalibacterium (2/11) (Table 3).

## Discussion

While some evidence has indicated the promise of efficient pathogen detection of suspected CNS infections using HTS, the practical clinical application of the technique remains understudied. The potential of HTS in microbial pathogen detection is underscored by advantages unique to the platform, including “hypothesis free” testing that does not rely on patient details or microbial knowledge, identification of varied strains of target pathogens, and ability to detect non-culturable or slow-growing organisms that may not be detected on traditional modalities. However, one of the most challenging aspects of HTS as a clinical tool is in the interpretation of data. HTS produces a large amount of complex data, requiring high-quality bioinformatics pipelines. Because of the untargeted nature of HTS, background interference must be filtered out, including highly repetitive nucleotide sequences, poor-quality and redundant sequences, and human host data.^13^ About 98% of CSF sequencing data maps to the human genome and must be removed.^14^ Additionally, patient samples must be handled with care to prevent cross-sample and environmental contamination.^15,16^ VirCapSeq-VERT and BacCapSeq methods have advantages over traditional HTS methods by, 1] Increasing sensitivity by enriching viral and bacterial read up to 10–1000 folds; 2] reducing host reads in final outcome by target enrichment for virus and bacteria, 3] reduction of required sequencing depth many folds and cost/sample.

Within our study, HTS detected pathogens in a considerable portion of our suspected CNS infection group (42%). However, a large majority of these reads were for commensal organisms and potential contaminants that have been studied previously.^15,16^ While it may appear that patients without definitive microbiological diagnosis who met criteria set by Brighton Criteria for meningoencephalitis (group 2) had a higher rate of pathogen detection, many of the pathogen reads require deeper analysis, contextualization, and interpretation. For example, we identified the presence of *Herbaspirillum* in the CSF of 2 patients based on the number of reads. Despite high read counts, the literature indicates that *Herbaspirillum* is typically a benign bacteria usually found in soil, but has been shown to be present in molecular biology grade water and laboratory reagents used in PCR and DNA extraction kits.^17^ While the sensitivity of this technique enables scientists to detect rare organisms with high human host backgrounds, environmental and reagent contamination is similarly detected and may be potentially misinterpreted. When there are low amounts of nucleic acid input, which is typically the case when sequencing CSF, contaminants tend to be overamplified, leading to high number of read counts of non-causative specimens.^18^ Although there are advanced molecular techniques that can address this hurdle, it is unlikely these methods would be used in a clinical setting.^13^

Similar to how traditional clinical test results require clinical context, potential pathogens determined through HTS are also enhanced by clinical context. In a previous study evaluating the use of HTS in clinical sequencing, a ‘clinical microbial sequencing board’ was established by experts in the field (neurology, ID, HTS, lab specialists) to discuss the data produced through HTS in the context of the clinical features of the case.^19^ In our patient sample, we selected patients with clinical suspicion for CNS infection, and thus were able to interpret the potential significance of the HTS reads in context of the clinical picture.

This study had several limitations. The primary limitation was the variation in time between sample collection and sequencing initiation among samples. Between samples, there was not a standardized time in which HTS was utilized for each case, and in several cases HTS was performed as a final measure for potential etiologic diagnosis. Given that pathogens may be undetectable in CSF in delayed stages of illness and CNS symptoms may appear only in post-acute period of infection during which genetic detection is not always possible, the utility of HTS as an early primary diagnostic tool is not fully captured in our study. This is especially illustrated in our study by samples in Group 1 diagnosed by serological tests (i.e. Lyme). Given these antibody tests may yield positive results in later phases of disease at which nucleic acid is degraded, sequencing may not be as useful when relying on serologies. This was a retrospective study in which CSF was taken via lumbar puncture and frozen before being sent to the laboratory. Nucleic acid degradation as a result of prior freeze-thaw steps may produce shorter, sparser sequencing reads which make it difficult to identify overlapping regions that allow de novo assembly into longer contiguous sequences, consequently leading to lower sensitivity of HTS.^20,21^ In this study, we utilized VirCapSeq-VERT, a technology with probes targeting viral taxa known to infect vertebrates, as well as BacCapSeq, which contains probes covering bacterial species including all known pathogens infecting humans.^6,7^ As a result, we were unable to detect potential fungal pathogens. Additionally, all of the sequences in this study underwent homology searches against the entire GenBank nucleotide database using MegaBLAST followed by NCBI BLASTx, which is a widely used approach.^21^ Other studies in non-clinical settings have utilized limited-target approach pipelines that can account for novel pathogen detection and aid in the interpretation of sequencing data. Our study analyzed the use of these techniques in a very small cohort of patients (34), which could also lead to potential error and variability within our results, along with significantly limiting the generalizability of the results. Lastly, the platforms utilized in this analysis were initially published upon in 2015 and 2018. Since then, additional technologies have been developed and investigated, however the efficacy of these more recent techniques is not reflected in our study.

While there are many potential applications of HTS, there are caveats that may hinder its ability to aid in the diagnosis of CNS infections in clinical settings. These include the cost, institutional capacity, false positive reads as a result of contamination, timing of sample collection, sample degradation, and a lack of clear criteria for diagnosis. HTS methods and workflows must be standardized. The establishment of a validated and universal bioinformatics pipeline would also aid in the clinical application of HTS. An a priori database of common reagent contaminants, in published literature as well as those seen in specific laboratory environments, should be established to compile a list of common contaminants to further aid in the interpretation of HTS results. Overall, HTS showed limited promise for pathogen identification in presumed CNS infectious diseases in our small sample. Larger-scale prospective studies should be conducted to fully assess the clinical value of this novel technique and improve its potential incorporation into clinical guidelines.

## Figures and Tables

**Table 1:** Clinical Vignettes of Group 1 (Definitive CNS Infection)

Clinical Vignette	Immune Status	Relevant PMH	Relevant Neuroimaging	Original Testing Modality	Identified Pathogen	Result on VirCapSeq-VERT and BacCapSeq in CSF
67F with progressively worsening altered mental status, right gaze deviation, and concern for possible seizure	Immunocompromised	Rheumatoid Arthritis Colonic perforation s/p recent high dose steroid & rituximab infusion	MRI brain: punctate acute cortical infarct in left inferior parietal lobe; mild sulcal hyperintensity in bilateral frontoparietal regions	CSF PCR	Herpes Simplex Virus-2 (HSV-2)	HSV-2
52M with delirium, low-grade fevers, generalized weakness, and L gaze preference s/p receiving immunotherapy	Immunocompromised	Stage IV diffuse large B-cell lymphoma undergoing CART therapy	MRI Brain: scattered nonspecific T2/FLAIR white matter hyperintensities	CSF PCR	Human Herpes Virus - 6 (HHV-6)	HHV-6B Pseudomonas
62F with painful vesicular rash of the R thigh and genital area, weakness of R leg, and numbness of the R lower leg	Immunocompromised	Primary-progressive Multiple Sclerosis Autologous stem cell transplant	MRI Spine: Diffuse spinal cord atrophy with probable scattered hyperintense foci of parenchymal signal. is consistent with chronic changes of multiple sclerosis. No abnormal cord enhancement.	CSF PCR	Varicella Zoster virus (VZV)	GB Virus C/ Human Pegivirus
31M with altered mental status, fever, seizures, and right leg rash	Immunocompromised	HIV (CD4 38; VL 37) Mycobacterium Avium Complex lymph node infection	CTH: new parenchymal hemorrhage anteromedial left temporal lobe and basal ganglia with intraventricular extension into left lateral ventricle.	CSF VZV Total and IgM	VZV HIV	VZV Torque Teno Virus
60M with altered mental status, vomiting, worsening headache, fever, and weight loss	Immunocompromised	IgA nephropathy Lymphocytic tubulitis Cytomegalovirus viremia End Stage Renal Disease s/p deceased donor renal transplantation x2	MRI Brain: FLAIR hyperintensity over leptomeningeal surface of brain; abnormal diffusion signal intensity in the dependent portion of the lateral ventricle consistent with debris in those ventricles.	CSF Cryptococcal antigen*	Cryptococcus neoformans	Negative *
68F with progressive subacute gait instability and dysarthria following recent decrease in immunosuppressant dose	Immunocompromised	Sjogren’s Disease Anti- synthetase syndrome Liver transplant candidate	MRI Brain: Patchy foci of abnormal signal and enhancement in the cerebellum greater than cerebrum with associated cerebellar volume loss	CSF PCR	JC Virus	Negative
48M with acute flaccid paraparesis and sensory loss of bilateral lower extremities	Immunocompetent	None	MRI brain: Mild parenchymal volume loss in parietal regions	CSF PCR	HSV-2	Negative
25F with progressive left leg numbness, headache, and neck pain/stiffness, arthralgia, fatigue, and pruritic lesions on the right arm and right thigh	Immunocompetent	None	MRI brain: multiple foci of T2/FLAIR hyperintensity in periventricular and subcortical white matter frontal horns; There is a well-circumscribed relatively bright signal intensity localized to the sphenoid sinus posteriorly and to the right most likely a small mucous retention cyst in the sphenoid sinus	Western blot (blood) IGM+; Borrelia C6 Peptide Ab+	Borrelia burgdorferi	Negative**

The clinical history including age, sex (male-M, female-F), presenting symptoms, immune status, notable past medical history (PMH), and neuroimaging data and lumbar puncture data was reviewed for patients with definitive CNS infection (Group 1). The original testing modality is stated alongside the HTS CSF reading

* Appropriately negative as not included in VirCapSeq-VERT or BacCapSeq assays

** Appropriately negative as pathogen detected by serological testing

**Table 2:** Clinical Vignettes of Group 2 (Meeting Brighton Criteria)

Clinical vignette	Immune status	PMH	Imaging	Clinical suspicion	LP Profile	Pathogen 1 (# of reads)	Pathogen 2 (# (reads)
50F with progressive ataxia, myoclonus, opsoclonus, neck stiffness, headache, photophobia, nausea, vomiting, and altered mental status >24 hrs	Immunocompetent	NMDA encephalitis (given rituximab) Migraine	MRI Brain: confluent diffuse T2/FLAIR hyperintensity involving white matter in both cerebral hemispheres and lesser extent pons	Postinfectious or paraneoplastic Opsoclonus myoclonus syndrome 2/2 Lyme	RBC: 1000/25* WBC: 15/13 Diff: 92% lymph Glucose: 59 Protein:55	Herbaspirillum (23,270)	
21M with headaches, photophobia, and 2 week history of new onset seizures	Immunocompetent	None	MRI brain w/ and w/o contrast and MRI of the Cervical and Thoracic spine were unremarkable	Resolving viral meningitis or treated bacterial meningitis vs. autoimmune	RBC:15/7 WBC: 72/67 Diff: 93% lymph Glucose: 55 Protein: 76	Bacteroides (288,913)	Faecalibacteriu (54,999)
57F with altered mental status >24 hrs, headache, transient R face/arm numbness and progressive confusion and decreased verbal output	Immunocompetent	Ulcerative Colitis	MRI brain: abnormal FLAIR signal intensity within sulci over convexity c/f acute meningitis; mild diffuse cerebral swelling	B12 deficiency vs. meningitis	RBC: 1220/449 WBC: 11/8 Diff: 55% neutrophils Glucose: 69 Protein: 52	Staphylococcus (129,309)	Neisseria (95,062)
51M with altered mental status >24 hrs, 2 days of word finding difficulty, malaise, chills, intermittent fevers with reduced oral intake and several weeks of diarrhea	Immunocompetent	Recent elective circumcision under general anesthesia in Dominican Republic	MRI brain & CTH: Normal	CNS viral meningoencephalitis	RBC: 4/0 WBC: 6/4 Diff: 47% neutrophils Glucose: 70 Protein: 60	No positive result	No positive resu
61M with altered mental status, fever, lethargy receptive aphasia And multiple recent admissions for potential aseptic meningitis	Immunocompetent	Recurrent aseptic meningitis (Rituxan) Migraine Atrial fibrillation (not on anticoagulation)	MRI brain: restricted diffusion in the left insula, left temporal lobe, left parietal lobe, and left occipital lobe as well as cortical/subcortical diffusion restriction in the posteromedial parieto-occipital lobe and associated curvilinear enhancement and FLAIR signal abnormality	Autoimmune encephalomyelitis	RBC: 1574/4 WBC: 14/1 Diff: 91% neutrophils Glucose: 124 Protein: 172	No positive result	No positive resu
60M with altered mental status > 24hrs, fluctuating confusion, gait unsteadiness, dizziness, left sided weakness, lethargy, and headache	Immunocompetent	Recent flu-like illness	Unremarkable	Viral encephalitis	RBC: 188/3 WBC: 13/35 Diff: 73% lymph Glucose: 70 Protein: 50	Leclercia (50,820)	Burkholderia (35,448)

The clinical history including age, sex (male-M, female-F), presenting symptoms, immune status, notable past medical history (PMH), neuroimaging data and lumbar puncture data was reviewed for patients meeting Brighton criteria (Group 2). The pathogen reads from HTS are provided in this clinical context. Protein and glucose are measured in mg/dL; red blood cell (RBC) in uL;

* RBC and WBC counts denoted tube 1/tube 4

**Table 3:** Clinical Vignettes of Group 3 (Not meeting Brighton Criteria)

Clinical vignette	Immune status	PMH	Imaging	Clinical Suspicion	LP Profile	Pathogen 1 (# of reads)	Pathogen reads)
33M with dysarthria, rightsided weakness, and pseudobulbar affect	Immunocompetent	Polio in childhood	MRI Brain: New foci of diffusion restriction with associated T2/FLAIR hyperintensity and enhancement in the left greater than right basal ganglia and thalami. Two additional foci of FLAIR hyperintensity and enhancement but without diffusion restriction in the right medial temporal lobe and right dorsal mid brain.	Autoimmune (Behcet’s/MS analog) vs infectious	RBC 0/0* WBC 180/ 251 64% neutrophil Glucose: 58 Protein: 84	No positive result	No positive
29F with recurrent seizures, right cranial nerve palsies, right-sided weakness, leftsided hyperreflexia, confusion, and vesicles in right ear	Immunocompetent	Progressive inflammatory neuropathy	MRI brain: Right temporal horn is slightly larger than left. Otherwise unremarkable without acute pathology.	Viral encephalitis	RBC: 3/3 WBC: 42/32 Diff: 83% neutrophil Glucose: 65 Protein: 40	No positive result	No positive
33F with fever, headache, worsening vision, difficulty walking, fevers, weakness, and lethargy	Immunocompromised	Cystic Fibrosis; Lung Transplant; Bronchiolitis obliterans syndrome; Squamous Cell Carcinoma	MRI Brain: mild increased T2 signal distal bilateral optic nerves/sheath; Adenoid FLAIR hyperintensity bilateral optic tracts MRI spine: mild hyperenhancement noted along several nerve roots in the lower thoracic spine which is nonspecific but concerning for arachnoiditis/spinal meningitis in context of intracranial findings	Viral meningitis	RBC: 179/172 WBC: 2/2 Diff: 97% lymph Glucose: 55 Protein: 52	Hepatitis E virus (58819)	
55F with altered mental status, fever, encephalopathy, subacute behavioral changes, and memory impairment	Immuno-competent	Sjogren’s Disease Latent TB+	MRI brain- Numerous small enhancing parenchymal lesions including in the left frontal lobe, left temporal lobe, left periventricular region, left genu of the corpus callosum, septum pellucidum and right posterior limb of the internal capsule with appearance concerning for CNS lymphoma.	CNS lymphoma	RBC:0/0 WBC: 8/7 Diff: 100% lymphocyte Glucose: 80 Protein: 20	No positive result	No positive
52F with several month history of anxiety, paranoia, and visual hallucinations along with acute presentation of acute jerking movements of arms, decreased speech, and weakness	Immuno-competent	Systemic Lupus Erythematosus; Recurrent HSV; Hashimoto’s thyroiditis	There is increased FLAIR sulcal hyperintensity diffusely. This could be related to leptomeningeal infectious inflammatory process, leptomeningeal blood products, high FiO2 or propofol administration	Autoimmune encephalitis	RBC: 1000/11 WBC: 11/8 74% lymph Glucose: 48 Protein: 195	No positive result	No positive
68F found unresponsive in bed at home, with emesis on pillow, metabolic disarray, altered mental status, and fever	Immuno-competent	Multiple episodes of altered mental status 2/2 urinary tract infection	Multifocal bilateral areas of restricted diffusion, T2 FLAIR hyperintensity with both cortical and white matter involvement and sparing of basal ganglia/thalami. There is involvement of the hippocampus.	Viral encephalitis	RBC:475/75 WBC:62/35 Diff: 95% neutrophil Glucose:62 Protein:56	No positive result	No positive
40F with diplopia, altered mental status, weight loss, weakness, and fevers,	Immune-compromised	HIV/AIDS (CD413, VL 460 copies/mL) not on antiretroviral therapy; Mycobacterium Avium Complex	MRI Brain: Mildly decreased conspicuity of multiple subcortical and deep white matter high T2/FLAIR lesions throughout bilateral supratentorial white matter, with stable lesions in bilateral thalami. New abnormal high T2/FLAIR signal lesions in left hippocampus and cerebellum. Weak CSF FLAIR suppression along the cortical sulci indicating a proteinaceous content.	PML BM	RBC: 0/0 WBC: 4/6 Diff: 51% lymph Glucose:56 Protein:32	No positive result	No positive
58M with tremors and altered mental status, L facial droop, L sided weakness	Immuno-competent	Bipolar 1 Disorder	MRI brain: Bilateral basal ganglia T2/FLAIR hyperintensity with no associated enhancement. abnormal FLAIR signal intensity which is bilateral and relatively symmetric involving the caudate head, globus pallidus, and the putamen. The involvement is slightly more prominent on the right than the left side.	Limbic encephalitis	RBC: 0/0 WBC: 2/1 60% lymph Glucose: 65 Protein: 26	No positive result	No positive
44F with 1 month of vertigo and hearing loss along with pain in the right side of her head, dizziness, nausea, and vomiting.	Immune-compromised	HIV (CD4 355, VL 52.8k, question ARV compliance )	MRI brain: abnormal area of FLAIR hyperintensity in the right frontal region 4–5 mm in size within a sulcus with associated minimal enhancement at that level ; Subtle linear FLAIR hyperintensity along the eighth cranial nerve on the right side in comparison to the left with a possible linear enhancement at that level	Viral meningitis	RBC: 19/26 WBC:47/42 Diff: 82% lymph Glucose: 49 Protein: 39	Human immunodeficiency virus 1 (67,3339)	
75F with altered mental status, right arm weakness, and aphasia, post traumatic neuralgia,	Immuno-competent	Recent herpes infection.	MRI Brain: No evidence of acute infarct, hemorrhage or mass effect. Mild chronic microvascular ischemic change in cerebral volume loss	Vasculitis VZV	RBC: 8000 WBC: 49 Diff: 52% lymph Glucose: 70 Protein 46	No positive result	No positive
68M with 1 week of anesthesia in perianal area, incontinence, and worsening sensation on left torso.	Immune-compromised	Liver Transplant on tacrolimus; Lymphoma; Laminectomies T5-T9	MRI TS: Stable fluid collection within posterior soft tissues in the area of prior laminectomy from T4-T9, with thick surrounding peripheral enhancement, unchanged. Findings most consistent with a seroma	Fungal infection	RBC: 11/0 WBC: 10/9 Diff: 92% lymph Glucose: 77 Protein: 102	Asticcacaulis (12,431)	
69M with altered mental status, difficulty walking, difficulty speaking and myoclonus 1 week after flu symptoms	Immuno-competent	None	MRI Brain: Small focus restricted diffusion posterior left lentiform nucleus/posterior limb of the left internal capsule suggestive of acute to subacute infarct. Clinical correlation advised; Chronic lacunar infarct left thalamus and adjacent corona radiata	Encephalitis Transient PRES seizure	RBC: 20/0 WBC: 1/1 Diff: 60% monocytes Glucose: 52 Protein: 81	No positive result	No positive
62F with nausea, vomiting, and first seizure of life.	Immuno-compromised	Lung cancer with CNS metastasis	MRI Brain: Localized area of abnormal FLAIR signal intensity in the subcortical white matter at the white-gray matter junction in the left posterior frontal region with no mass effect, no diffusion abnormality, and no abnormal enhancement this area of presumed vasogenic edema in the white matter may be sequela of recent seizure from the adjacent cortex	PRES AE	RBC: 8000/1000 WBC: 1/1 Diff: 60% lymphs Glucose: 67 Protein: 35	No positive result	No positive
66F with increased confusion, vertical gaze palsy and gait instability who was found to have distal shunt malfunction and development of ventriculitis and sustained hydrocephalus following revision.	Immuno-competent	Normal Pressure Hydrocephalus Multiple surgical shunt placements	CTH: Interval decrease in the size of the left lateral ventricle with interval increase in the size of the right lateral ventricle as detailed.	Cerebral ventriculitis	RBC: 1000/4000 WBC: 9/2 Diff: 68% lymph Glucose: 39 Protein: 155	Bacteroides (15,637)	
36F with difficulty walking, bilateral leg weakness, numbness/tingling in RLE, spasticity	Immuno-competent	C1-C2 and T4- 5 fracture; Spinal cord reconstruction Recent upper respiratory infection	MRI TS: Relatively small thoracic spinal cord, with smooth tapering from normal caliber around C7-T1. 8 mm focus of T2 signal hyperintensity within the central cord at T3-T4 is nonspecific likely represents a focal syrinx. No associated abnormal enhancement.	Transverse myelitis	RBC: 5000/33K WBC: 11/36 Diff: 70% neutrophils Glucose: 57 Protein: 43	No positive result	No positive
69F with altered mental status, acute headache, vomiting, lethargy, and difficulty communicating	Immuno-competent	Recent pneumonia	Unremarkable	Bacterial meningitis	RBC 125/127 WBC: 9088/5100 Diff: 87% neutrophils Glucose: 55 Protein: 576	No positive result	No positive
13F with headache, worsening upper extremity weakness with numbness, tingling, and soreness	Immuno-competent	Cervical neuropathic pain	MRI CS: Persistent patchy spinal cord signal abnormality centered within the gray matter right greater than left from C2-C3 through C3-C4 and questionably at C1-C2 and C6; New corresponding patchy cord enhancement from the levels of C2-C3 through C3-C4.	Transverse myelitis	RBC: 6000/1000 WBC:8/2 50% lymph Glucose: 61 Protein :34	No positive result	No positive
43M with sudden onset left sided face and eye twitching followed by left sided shaking followed by left sided hemiparesis for approximately twelve hours in the context of recent diagnosis of miliary TB on LIPE therapy	Immuno-compromised	Alcoholic cirrhosis; Miliary tuberculosis on LIPE with right parietal tuberculoma	MRI: Interval increase in size of the high right frontoparietal ring- enhancing lobulated/septated lesion and progressive surrounding vasogenic edema and restricted diffusion.	Brain abscess 2/2 TB/fungal	RBC: 1/0 WBC: 1 /1 Diff: 84% lymph Glucose: 79 Protein: 22	Bacteroides (38,003)	Facealibac (32,498)
73F with altered mental status, aphasia, and new onset focal status epilepticus following liver transplantation	Immuno-compromised	Neuroendocrine tumor; Primary Biliary Cholangitis; Liver transplant (on tacrolimus)	MRI: Enhancing T1 hyperintense and T2 heterogeneous hyperintense mass in the right cerebellopontine angle extending from the right lateral pons into the right internal auditory canal measuring up to 2.2 cm is most consistent with a vestibular schwannoma. There is mild mass effect on the right middle cerebellar peduncle. There is no associated hydrocephalus.	Post transplant AE vs bacterial meningitis	RBC: 39/1 WBC: 1/9 Diff: 89% neutrophils Glucose: 66 Protein: 58	Rothia (15,503)	
8M with headache, emesis, and abnormal gait for 6 months	Immuno-competent	None	MRI: There are several T2/FLAIR hyperintense foci in the bilateral frontoparietal white matter, largest in the right parietal subcortical white matter measuring 1 cm. These are indeterminate. No abnormal cord signal or enhancement.	Migraine vs ADEM vs postinfectious encephalitis	RBC: 0 WBC: 1 68% lymph Glucose: 64 Protein: 17	Klebsiella (40,286)	

The clinical history including age, sex (male-M, female-F), presenting symptoms, immune status, notable past medical history (PMH), neuroimaging data and lumbar puncture data was reviewed for patients not meeting Brighton criteria (Group 3). The pathogen reads from HTS are provided in this clinical context. Protein and glucose are measured in mg/dL; red blood cell (RBC) in uL;

* RBC and WBC counts denoted tube 1/tube 4
